# The Effect of Embedded Nanoparticles on the Phonon Spectrum of Ice: An Inelastic X-ray Scattering Study

**DOI:** 10.3390/nano13050918

**Published:** 2023-03-01

**Authors:** Alessio De Francesco, Luisa Scaccia, Ferdinando Formisano, Eleonora Guarini, Ubaldo Bafile, Dmytro Nykypanchuk, Ahmet Alatas, Mingda Li, Scott T. Lynch, Alessandro Cunsolo

**Affiliations:** 1 CNR-IOM & INSIDE@ILL c/o Operative Group in Grenoble (OGG), F-38042 Grenoble, France; 2 Institut Laue-Langevin (ILL), F-38042 Grenoble, France; 3 Dipartimento di Economia e Diritto, Università di Macerata, Via Crescimbeni 20, I-62100 Macerata, Italy; 4 Dipartimento di Fisica e Astronomia, Università di Firenze, Via G. Sansone 1, I-50019 Sesto Fiorentino, Italy; 5 Consiglio Nazionale delle Ricerche, Istituto di Fisica Applicata ”Nello Carrara”, Via Madonna del Piano 10, I-50019 Sesto Fiorentino, Italy; 6 Brookhaven National Laboratory-National Synchrotron Light Source-NSLS II, P.O. Box 5000, Upton, NY 11973, USA; 7 Argonne National Laboratory, Advanced Photon Source, P.O. Box 5000, Upton, NY 11973, USA; 8 Department of Nuclear Science and Engineering, Massachusetts Institute of Technology, Cambridge, MA 02139, USA; 9 Department of Physics, University of Wisconsin—Madison, 1150 University Avenue, Madison, WI 53706, USA

**Keywords:** inelastic X-ray scattering, phonon propagation, nanoparticles, model choice, Bayesian inference, terahertz phononics

## Abstract

As a contribution to the ongoing effort toward high-frequency sound manipulation in composite materials, we use Inelastic X-ray Scattering to probe the phonon spectrum of ice, either in a pure form or with a sparse amount of nanoparticles embedded in it. The study aims at elucidating the ability of nanocolloids to condition the collective atomic vibrations of the surrounding environment. We observe that a nanoparticle concentration of about 1 % in volume is sufficient to visibly affect the phonon spectrum of the icy substrate, mainly canceling its optical modes and adding nanoparticle phonon excitations to it. We highlight this phenomenon thanks to the lineshape modeling based on a Bayesian inference, which enables us to capture the finest detail of the scattering signal. The results of this study can empower new routes toward the shaping of sound propagation in materials through the control of their structural heterogeneity.

## 1. Introduction

At non-zero temperatures, atoms in a crystal experience oscillations around the respective lattice positions. At variance with gases, where atomic motions are erratic, such oscillations are highly interconnected due to the tight microscopic bonds. Thus, a single atom’s displacement from its lattice (equilibrium) position can be transmitted to successive neighbors, thus forming propagating lattice distortions called phonons [1]. In non-conductive materials, terahertz acoustic phonons are the leading conveyors of the heat flow. The control of the terahertz sound propagation is thus essential for developing a new class of metamaterials with tailored thermal properties [2]. In recent years, this circumstance has stimulated a vivid interest in implementing heat flow management based on the manipulation and steering of high-frequency sound propagation [2]. The so-called phononic crystals (PC) are ideal devices for this scope due to the periodicity of their structure, which is designed to interfere with sound waves in a given frequency band, causing them to slow down, deviate, or get trapped in a so-called phononic gap. Although the development of a PC active from kilohertz to megahertz frequencies rapidly evolves toward maturity, the terahertz domain remains almost uncharted. An alternative pathway to control the terahertz phonon propagation foresees the use of acoustic metamaterials instead [3], in which sound propagation is controlled via local resonators which absorb acoustic waves in specific frequency windows. The periodical arrangement is optional for these devices, although it might be an asset. Finally, one can control the sound propagation by simply introducing some elastic heterogeneity. Along this line, in a series of Inelastic X-ray Scattering (IXS) measurements on nanoparticle (NP) suspensions in liquids [4,5,6,7], we observed that NPs, even in a sparse concentration, can enhance the acoustic damping of the host liquid. One of the questions still left open by these studies is whether embedded nanoparticles can have a visible impact on the propagation of excitations living longer than collective modes in a liquid, as phonon-like modes in a polycrystalline material. To shed some light on this hypothesis, here we decided to use IXS to characterize the phonon response of a frozen gold nanoparticle (AuNP) aqueous suspension and compare it with the pure polycrystalline ice Ih investigated in our recent work [8]. We recall here that the phonon dispersion of Ih was previously studied by IXS [9,10], INS [11], and the two techniques combined [12].

## 2. Materials and Methods

**Figure 1:**
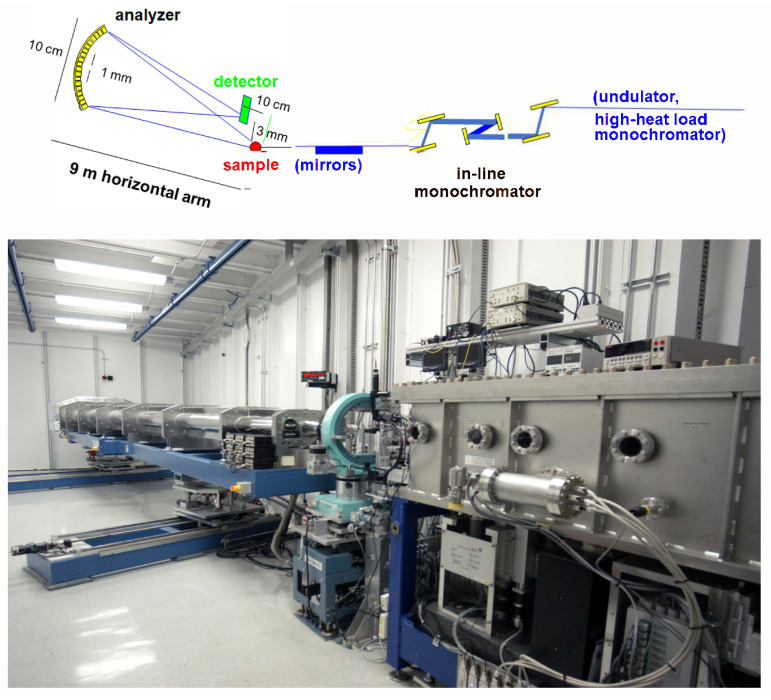
The upper panel represents a schematic diagram of the used instrument, while the lower one includes a picture of the sample spectrometer hutch.

The two icy samples were at the same temperature (*T* = 225 K), and their IXS spectra were collected in the same exchanged wavevector *Q* range (3–21 nm${}^{-1}$). Measured spectral shapes were approximated by a multiple phonon excitation model profile in which the most plausible number of phonon modes was determined through a Bayesian analysis of measured spectra, as described in Refs. [13,14]. The measurements were executed using the high-resolution *IXS* beamline Sector 30 of the Advanced Photon Source at Argonne National Laboratory [15,16]. The spectrometer was operated with an incident beam energy of 23.7 keV, corresponding to the Si(12 12 12) backscattering reflection from the spherical analyzers. The scattering from the sample was energy analyzed by 9 independent analyzers mounted on the moving extreme of a spectrometer arm rotating horizontally around the sample axis and mutually separated by a constant *Q* offset, ΔQ = 2 nm${}^{-1}$. The energy analysis is implemented through the rocking of the crystals of the monochromator unit while keeping the geometry of the Si analyzer crystals fixed. Given the incident X-ray wavelength, λ, the angle between the incident beam and the spectrometer arm determines the *Q* value probed by each analyzer. Once the spectrometer is aligned, the energy resolution profile slightly varies for each analyzer, with an average resolution FWHM of about 1.2 meV. In Figure 1 is shown a sketch of the Sector 30 beamline. Further details on the spectrometer can be found elsewhere [15].

Au nanoparticles were synthesized by Nanopartz (https://www.nanopartz.com. accessed on 19 February 2023). They are spherical particles with a size accuracy of ±2 nm, a diameter of 15 nm, and a <4% polydispersity. Particle concentration in the water suspension is 1% in volume as estimated by an ultraviolet–visible spectroscopy measurement. They show structural properties similar to those of bulk gold, although their X-ray pattern features broader peaks due to their small crystalline domain size. After accomplishing the various measurements, we performed a post-experiment inspection of the suspension, observing that the latter preserved its integrity upon melting, with no sign of precipitation typical of liquid suspensions recovered after freezing-induced segregation.

**Figure 2:**
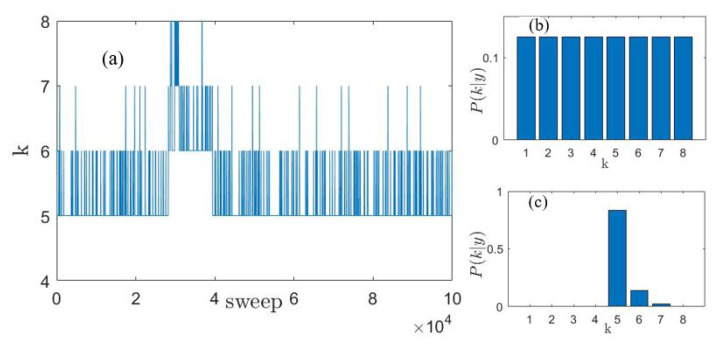
Traceplot (panel **a**) showing the model selected at each MCMC sweep, prior (panel **b**) and posterior (panel **c**) distribution for the number *k* of inelastic components in the spectrum of the AuNP suspension at *Q* = 21 nm${}^{-1}$.

One may wonder whether the sample was in a polycrystal form. Some facts persuaded us that this was indeed the case; these include the insensitivity of the scattering profile to sample orientation, the consistency in the position of ice phonon branches in the two explored samples, the disordered nature of the sample suggested by the dominating elastic peak, as well as the consistency of our results with those reported in the literature for both polycrystalline ice and water. The Bayesian inference method used here exploits an ad hoc crafted Markov Chain Monte Carlo [17,18] algorithm integrated with a Reversible Jump option [19]; it was successfully tested on Brillouin neutron scattering data of crystalline UFe${}_{2}$ [20] and successively applied to INS measurements on liquid gold [13] and silver [21], as well as to IXS [4,5,7,22] and neutron Spin Echo [23] measurements. For a detailed description of the used algorithm, we refer to these works, here only mentioning that, within this approach, the scattering profile, proportional to the structure factor S(Q,E) [24], was approximated by the sum of a central peak and a finite number of excitations. Namely,

(1) $$\begin{array}{ccc} S(Q,E) & = & A_{e}\left(Q\right)\delta \left(E\right)+\left[n\left(E\right)+1\right]\frac{E}{k_{B}T}\left\{\sum_{j=1}^{k}\frac{2}{\pi }A_{j}\left(Q\right){\mathrm{DHO}}_{j}\left(Q,E\right)\right\}\; \end{array}$$

where E=ℏω is the energy transferred from the probe particle to the target sample, δ(E) the Dirac delta function describing the elastic response of the system defined by an intensity factor $A_{e}\left(Q\right)$, $n\left(E\right)={\left(e^{E/k_{B}T}-1\right)}^{-1}$ is the Bose factor expressing the detailed balance condition, and the term in curly brackets represents *k* inelastic contributions. The latter are approximated by Damped Harmonic Oscillator (${\mathrm{DHO}}_{j}\left(Q,E\right)$) doublets [25] having amplitudes $A_{j}\left(Q\right)$ and

(2) $$\begin{array}{c} {\mathrm{DHO}}_{j}\left(Q,E\right)=\frac{\Omega_{j}^{2}\left(Q\right)\Gamma_{j}\left(Q\right)}{{\left(E^{2}-\Omega_{j}^{2}\left(Q\right)\right)}^{2}+4{\left[E\Gamma_{j}\left(Q\right)\right]}^{2}} \end{array}$$

where $\Omega_{j}\left(Q\right)$ and $\Gamma_{j}\left(Q\right)$ are the undamped energy and the damping coefficient of the *j*th DHO excitation, respectively. Notice that the number *k* of ${\mathrm{DHO}}_{j}\left(Q,E\right)$ excitations likely to appear in the spectrum and their shape coefficients are equally treated as adjustable parameters in the fitting routine. The Bayesian framework requires the specification of a prior distribution for each model parameter to account for any a priori information by integrating it in the inferential process in a consistent and sensible probabilistic way. Besides the prior distributions of all lineshape model parameters, defined in detail in Ref. [13], a prior distribution needs to be chosen, of course, for the number of peaks, *k*, as well. As a matter of fact, the superimposition of a number of inelastic modes to a model is probably one of the most critical points when attempting to model and interpret the density fluctuations spectrum, S(Q,E), of the system under investigation. This becomes especially critical when dealing with liquids or disordered systems or, as in the present case, with polycrystalline samples. In all these cases when multiple phonons (or pseudo-phonons, as sometimes they are called) are present, the different inelastic contributions might be hardly distinguishable, either because they could mutually overlap or due to resolution limitations. Furthermore, in fluid, liquid, or glassy systems, collective excitations have a much shorter lifetime than their counterparts in crystalline solids, and this translates into a significant broadening of spectral features, which challenges their detection. For this reason, we believe that, in the analysis of scattering data, the determination of a reliable and probabilistically sound value for *k* might be one of the crucial objectives of the inference. The Bayesian approach provides us with rigorous protection from two opposite risks: the overinterpretation of our results, using or abusing a redundant number of parameters in the fitting model, and the possibility, on the contrary, of missing some weak but nonetheless significant contribution to the spectral shape. For this reason, we assume *k* to be adjustable, with prior probability $p\left(k\right)=p_{k}$, for $k=1,\ldots ,k_{max}$, where $k_{max}$ (equal to 8 in the present work) is chosen to be an appropriate limit for the possible number of peaks. This prior can accommodate any level of belief on the actual value of *k*, from complete ignorance, by letting $p_{k}=1/k_{max}$, ∀k, to perfect knowledge, by assuming $p_{k}=\delta_{k,k_{0}}$, where $k_{0}$ is known with certainty to be the value of *k* and δ is the Kronecker delta function. As a consequence of our choice of a uniform prior, the posterior distribution of *k* depends on the data only and is not influenced by any physical prior knowledge of the phenomenon under study (see Ref. [26] to compare two different choices for *k* prior distribution). Figure 2 shows how the prior distribution (panel b) is modified by the experimental data to provide, after a certain number of steps (sweeps) of the MCMC-RJ algorithm, the posterior distribution (panel c) for the parameter *k*. Panel a displays the so-called traceplot for the number of inelastic components, which clearly illustrates how the algorithm jumps through the different values of *k*, changing the dimension of the model parameters hyperspace, each time a new value for the variable is accepted according to the prescription acceptation rule exploited [13].

To provide an accurate approximation of the measured spectrum, the model function in Equation (1) must be convoluted with the instrument resolution function R(Q,E) and a spectral background added to such a convolution. Explicitly,

(3) $$\tilde{S}\left(Q,E\right)=R\left(Q,E\right)⊗S\left(Q,E\right)+B\left(E\right)$$

where B(E) is expectedly a mildly linear *E*-dependent background intensity.

## 3. Discussion of Results

In general, dealing with frozen suspensions offers some advantages compared to their liquid counterparts due to the much better-defined spectral excitations. Still, their physical interpretation and link with microscopic vibrations remain a significant challenge.

**Figure 3:**
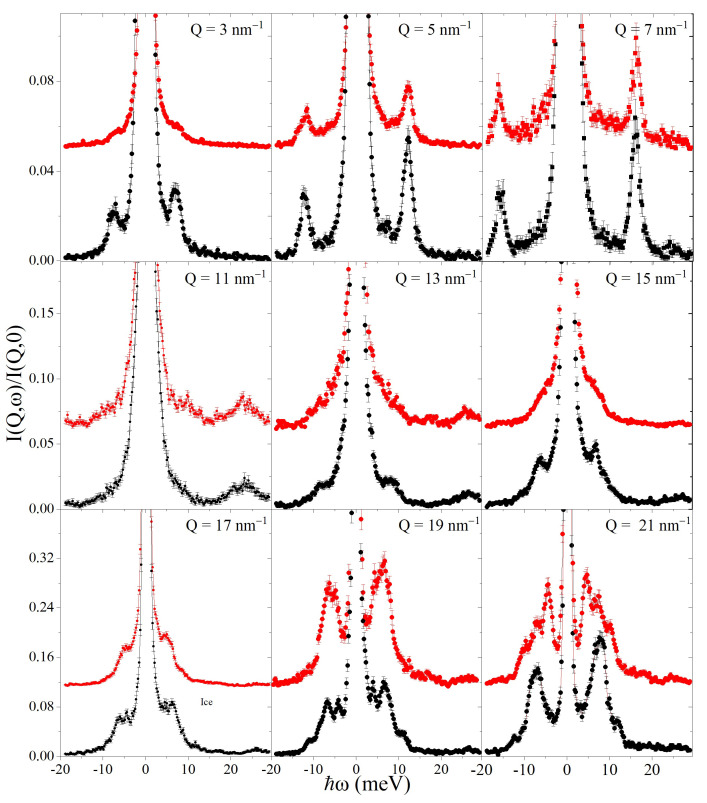
*IXS* spectra from the ice samples (black dots) at the indicated *Q* values are compared with those of the frozen suspension (red dots). Spectra are normalized to their maximum and represented in an expanded *y*-scale to emphasize the shape of the inelastic features. Spectra from the frozen suspension are vertically offset for clarity.

In Figure 3, the IXS spectra of the AuNPs frozen suspension are compared with those measured in the pure ice at the corresponding *Q* values. The lineshapes are normalized to their intensity maxima; furthermore, those from the suspension are vertically offset for clarity.

A few striking differences readily emerge between the scattering profiles from the two samples. For instance, at *Q* = 3 nm${}^{-1}$, 13 nm${}^{-1}$, 15 nm${}^{-1}$, and 17 nm${}^{-1}$, low-frequency inelastic features in the frozen suspension spectra are visibly smoother. At Q= 5 and 7 nm${}^{-1}$, they have a slightly smaller relative amplitude, while at 19 and 21 nm ${}^{-1}$, they have a differently structured profile. As inspected by the naked eye, these differences may appear more erratic than systematic, and the detailed effect of embedded AuNPs on the phonon response is hardly unraveled without further analysis. The likely emergence of Au phonons in the NP interior makes the scenario even more puzzling. In fact, despite the sparse NP concentration (less than 1% in volume), intense spectral features seem to stand out from the highest *Q* spectral profiles at about the energies expected for Au acoustic phonons as observed in previous measurements on a suspension of AuNPs in water [4] as well as if compared with the energies derived by the density of states computed for Au crystal [27]. A further discussion on the effect of embedded AuNPs in the shape of ice phonon spectra can be found in the Supplementary Material (see Figures S4–S7).

The rich variety of inelastic features challenges the lineshape modeling and the subsequent mode assignment. Indeed, measured spectra show a varied landscape of inelastic contributions gathering in a relatively limited range of energy. Occasionally, these modes overlap, becoming indistinguishable whenever separated by less than the resolution FWHM. Furthermore, upon varying *Q*, their shift can move back and forth in an unpredictable fashion, thus hampering our ability to trace their *Q*-evolution and distinguish them from neighboring excitations. A valid guiding criterion in this endeavor is a continuity check on the peak frequencies and amplitudes. Most importantly, a Bayesian inference is critical to unraveling this complex mapping, as it is grounded on experimental evidence, but, at the same time, it encompasses our relevant a priori knowledge of the physical problem at hand and, in particular, of the relevant literature. However, in the current study, we deliberately adopt an agnostic approach, given the higher complexity of the hybrid system under investigation. Moreover, given the undifferentiated analytic (DHO) account of all spectral excitations, a Bayesian inference, like any other inferential method, cannot be used to “label” the spectral features identified by assigning them to specific dispersion branches. Of course, it can even unravel underlying atomic vibrations less by associating them with specific atomic movements. This interpretative effort is, of course, entirely entrusted to the researcher’s judgment and knowledge, and, as such, it is prone to some arbitrariness.

**Figure 4:**
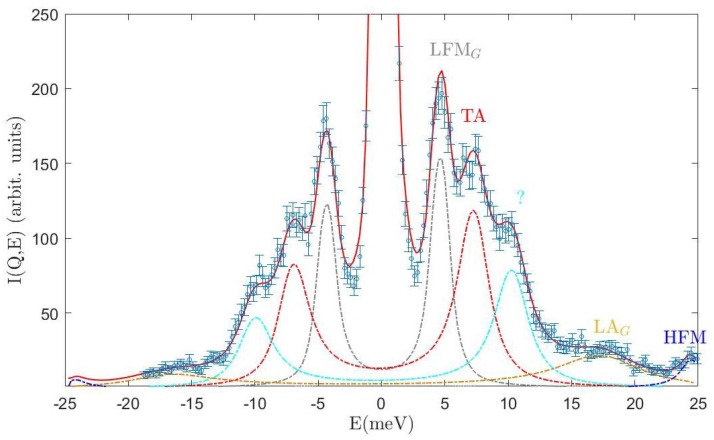
The spectrum of the frozen suspension at *Q* = 21 nm${}^{-1}$ is compared with best-fitting lineshapes and the individual lineshape components. The corresponding color acronym indicates each spectral mode’s assignment: LFM${}_{G}$ is a low-frequency mode associated to Au nanoparticles, TA (ice) transverse acoustic, LA${}_{G}$ Au longitudinal acoustic, HFM high-frequency (ice) mode (see text). The question mark labels a not assigned mode as discussed in the text. The spectrum is displayed in an expanded *y*-scale to emphasize the shape of the inelastic features.

**Figure 5:**
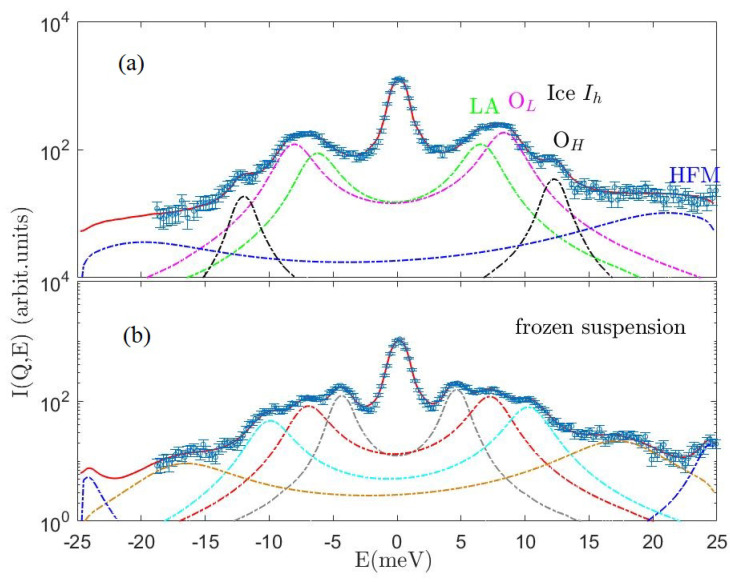
The *IXS* spectra of ice and the frozen aqueous suspension of the AuNPs at *Q* = 21 nm${}^{-1}$ in panels (**a**,**b**), respectively. Raw data are compared with best-fitting model lineshapes and individual spectral components, with the same color code as in Figure 4 for the AuNPs suspension yet in semilogarithmic plots. For the ice Ih spectrum, the two optical modes $O_{L}$ and $O_{H}$ are in magenta and black, respectively, and the mode LA is in green.

For these reasons, our phonon assignment might sporadically become questionable if multiple peaks fully overlap; however, the inferential method adopted makes us confident that our mode spotting and location is the one probabilistically most supported by the measurements. Clear examples of the mode overcrowding of some spectra are provided by Figure 4 and Figure 5, both referring to the *Q* = 21 nm${}^{-1}$ case.

When spectral features barely stand out from the spectral background or are partially obscured by neighboring peaks, a Bayesian inference becomes critical to assess their statistical significance. For instance, in Figure 5, this seems the case for both the longitudinal acoustic mode, LA, and the high-frequency mode, HFM. In Ref. [8], we ascribed the former mode to lattice vibrations involving the H${}_{2}$O molecules’ center of mass, i.e., the ordinary longitudinal acoustic mode, in view of its low *Q* propagation speed. Our interpretation of the latter mode is more uncertain; however, we noticed that its speed and the LA one scale with the square root of the ratio between the oxygen and H${}_{2}$O masses. This suggests that, at sufficiently low *Q*s, this mode relates to propagating vibrations involving the oxygen atoms. Spectral profiles are always dominated by lower-frequency modes, whose relative amplitude seems very sensitive to embedded NPs. Surprisingly, we find out that the optical branches and the LA of the ice Ih are strongly attenuated in the frozen suspension (see also Figure S1 of the Supplementary Material).

**Figure 6:**
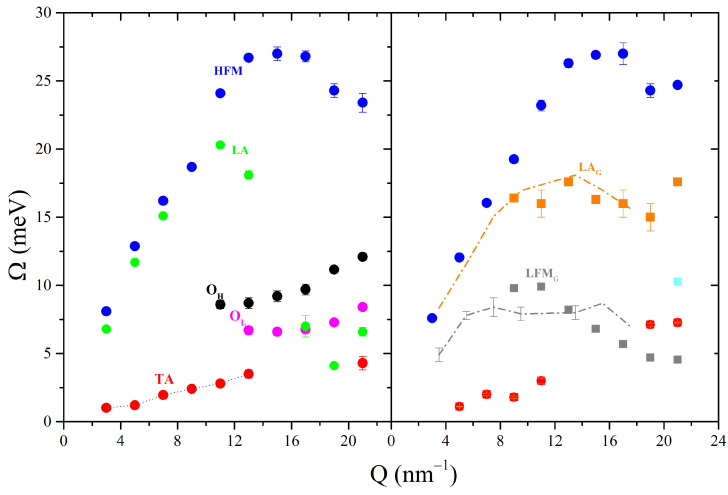
Left and right plot display, respectively, the dispersion curves detected in pure ice (dots) and in the frozen suspensions (squares). The dash-dotted orange line in the right plot represents the dispersion curve of the only (longitudinal acoustic) mode observable in liquid gold [13,28,29], while the corresponding gray line is the transverse Au phonon dispersion observed in our *IXS* measurement on a liquid AuNP suspension [4]. Each mode is associated with a color and labeled in either of the two plots with the acronyms of the same color, whose meaning is explained in the main text.

The model lineshapes, obtained as mentioned above, are well approximated by the model, as suggested by Figure 5, which compares the raw and best fit lineshapes of the two samples at the highest *Q* = 21 nm${}^{-1}$ value investigated. The number of excitations and relative amplitudes and positions varies in the two samples, thus suggesting some interference between the immersed NPs and density waves propagating throughout the icy hosting medium. The inspection of the dispersion curves in Figure 6 provides a more quantitative assessment of the role of NPs on the terahertz phonon modes of the icy substrate. As discussed in detail in a previous IXS work [8], the phonon dynamics of such a substrate in the 1 ÷ 21 nm${}^{-1}$, 0 ÷ 30 meV, dynamic range features five modes: a longitudinal acoustic one related to lattice vibrations of the molecular center of mass (LA) and a high-frequency mode (HFM) associated to the oxygen atoms alone, a transverse acoustic (TA) mode, and lower- and higher-energy optical modes, labeled as O${}_{L}$ and O${}_{H}$, respectively. Interestingly, the comparison of the dispersion curve in Figure 6 suggests that no signature of the optical branches and the LA of the ice Ih can be found in the frozen suspension. Not unexpectedly, we observe the emergence in the frozen suspension of two additional branches, which we ascribe to a longitudinal acoustic mode and a low-frequency phonon mode propagating in the NP interiors, here labeled as LA${}_{G}$ and LFM${}_{G}$, respectively. As mentioned above, this assignment rests on the favorable comparison in Figure 6 between the higher-frequency branch (orange squares) and the longitudinal acoustic mode of the liquid Au ([13,28,29], orange dash-dotted curve) and between the lower-frequency one (gray squares) and our measurement of a similar mode in a AuNP liquid suspension [4]. While the first comparison is evidence of an excellent agreement, the second has more significant uncertainty, owing to the limited statistical accuracy in our measurement on the liquid suspension. One can notice the presence of an additional cyan square symbol in the plot of the frozen dispersion curves; although the origin of that additional mode is unclear, the Bayesian analysis identified its presence in the *Q* = 21 nm${}^{-1}$ spectrum beyond ambiguity.

**Figure 7:**
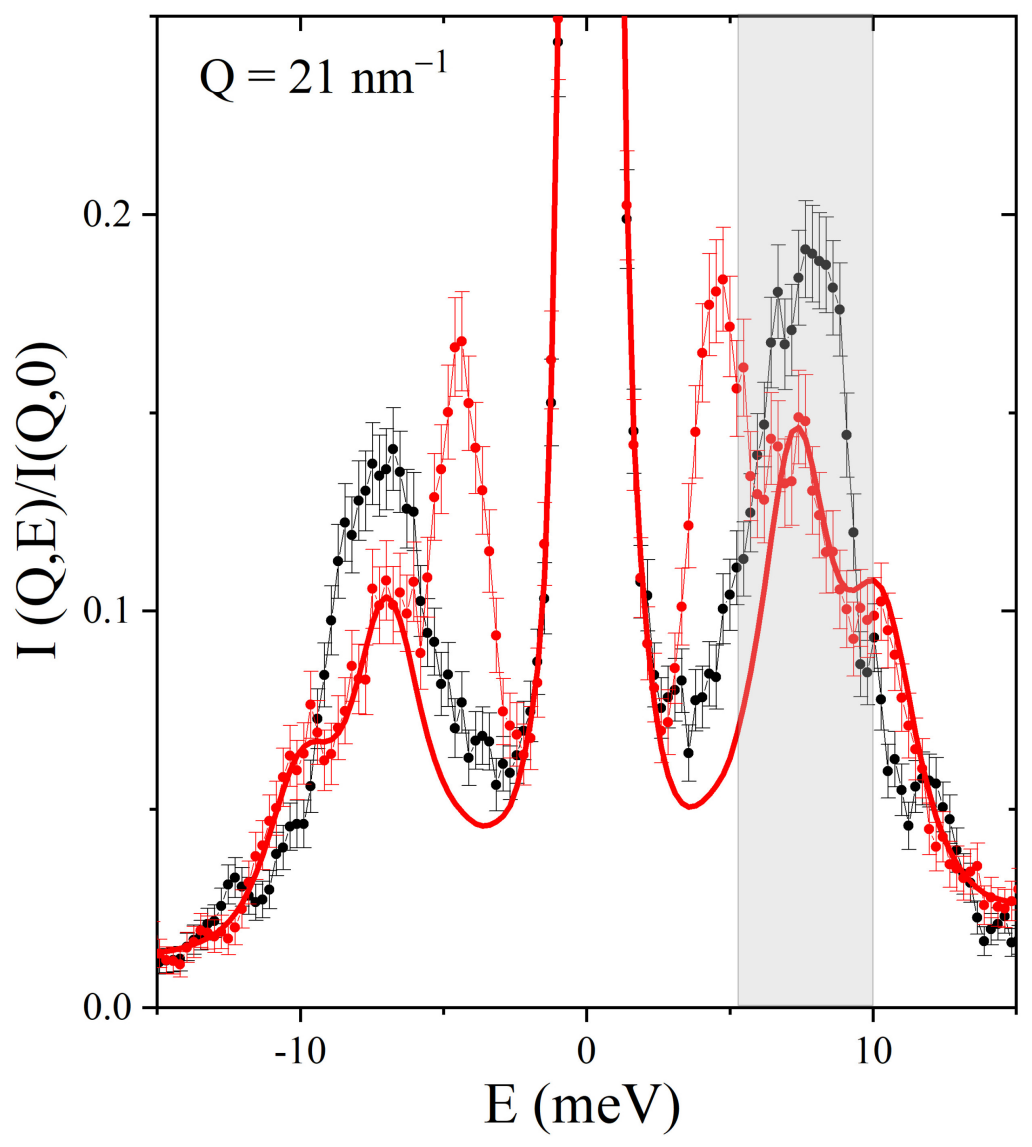
IXS spectra measured on polycrystalline ice (black dots) and AuNP frozen suspension (red dots) at *Q* = 21 nm${}^{-1}$ are compared in a restricted energy range after normalization to the respective maxima. The shadowed gray band covers the zone where the optical modes of ice are detected. The plot includes as a red line the best-fitting lineshape of the frozen suspension spectrum without its low-frequency DHO component after similar normalization.

In Figure 7, we finally consider one of the cases where the effect of the embedded nanoparticles leaves the most evident signature on the lineshape. There, the spectra measured in the two samples at *Q* = 21 nm${}^{-1}$ are compared to each other and with the best-fitting lineshape of the frozen suspension spectrum without its low-frequency DHO component (here assigned to the LFM${}_{G}$ mode). The comparison of the figures clearly shows that (1) the dominant inelastic feature of the ice spectrum containing the two optical modes O${}_{L}$ and O${}_{H}$ seems strongly attenuated in the suspension lineshape without the Au transverse contribution. (2) However, such a subtraction uncovers a peak located near the center of the gray band. We assign such a peak to the TA mode of water, which at these *Q* values largely overlaps in energy with the “optical doublet”; the circumstance that this doublet essentially submerges the TA mode explains why the latter can hardly be detected in pure ice spectra at these *Q*s.

## 4. The Central Role of Bayesian Analysis

A question may arise about the actual use of the Bayesian inference when dealing with spectra dominated by relatively sharp features and thoroughly investigated by the past and recent literature. However, the mentioned overlap between the phonon modes and the concurrent resolution limitations hamper a reliable discernment of differences and analogies in the spectral shapes under comparison. Most regrettably, it also exposes investigators to two opposite risks: (i) the “unauthorized assumption of novelty,” which causes investigators to favor unnecessarily complex and over-parametrized models, and (ii) the “confirmation bias”, which conversely prevents one from acknowledging the originality of the results at hand due to the excessive reliance on previous beliefs. An evidence-based approach, such as a Bayesian analysis, can successfully cope with these opposite biases inherent to human nature and typically mirrored by the inclusion of either an excessive or an insufficient number of excitations in the model.

**Figure 8:**
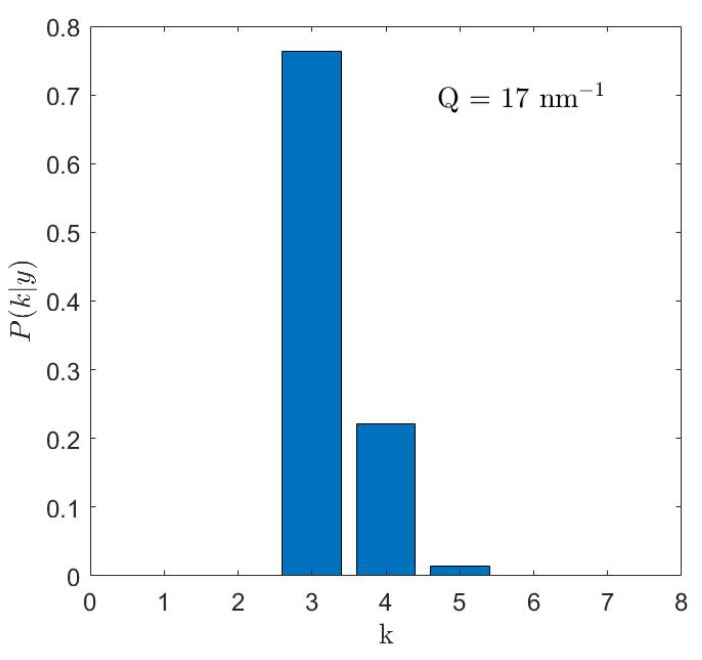
Simulated posterior distribution for the number, *k*, of inelastic components given the experimental data *y* for the frozen suspension of AuNPs at a momentum transfer of *Q* = 17 nm${}^{-1}$.

The adopted Bayesian method provides probabilistically grounded quantitative support to the choice of a given model. For instance, it can explicitly identify a certain number of excitations and rate their plausibility. There are situations in which the inelastic peaks partially overlap, and the number of modes rated as the most plausible by the Bayesian algorithm is correspondingly smaller (see Figure 8). Of course, as already stressed above, a Bayesian inference by itself cannot “give labels” to such excitations and can even less unravel the underlying atomic vibrations.

## 5. Conclusions

We have discussed the results of an IXS measurement on a dilute aqueous gold nanoparticle suspension below freezing and compared them with those previously obtained on polycrystalline ice. The comparison of the scattering signals from the two samples demonstrates that the embedded nanoparticles, despite their sparse concentration, visibly affect the spectral shape of the hosting medium, especially at the highest and lowest values of the exchanged momentum. Based on the outcome of a Bayesian inference-based lineshape analysis, we propose a consistent assignment of the phonon modes of the two samples under comparison, clearly emphasizing the influence of nanoparticles on the phonon response of the hosting fluid. Our phonon assignment, although thoroughly pondered, might be questioned in view of the pitfalls mentioned above; nonetheless, besides its overall self-consistency and scientific soundness is also the interpretation probabilistically most supported by the experimental outcome. Specifically, we observe that embedded nanoparticles cause the suppression of some phonon modes from the spectral shape and the parallel emergence of the two acoustic phonons propagating through the nanoparticle interior. A natural extension of this study would be replacing nanoparticles with more complex nano-objects as engineered mesoscale architectures, thus exploring the possibility of shaping the terahertz sound propagation through the structure’s design.

## Data Availability

The data presented in this study are available on request from the corresponding author.

## Supplementary Materials

The following supporting information can be downloaded at: https://www.mdpi.com/article/10.3390/nano13050918/s1, Figure S1: Comparison among IXS spectra measured on polycrystalline ice and AuNP frozen suspension at *Q* = 15 nm${}^{-1}$; Figure S2: Curve fit for the IXS spectrum of the AuNP frozen suspension at *Q* = 15 nm${}^{-1}$ for the optimal and the suboptimal solution as estimated by the MCMC-RJ algorithm; Figure S3: Posterior distribution functions for the excitation frequencies Ωj in the low energy range for the optimal and suboptimal solution as estimated by the MCMC-RJ algorithm; Figure S4: Comparison between the IXS spectra measured at Q = 19 nm${}^{-1}$ on the ice sample and the frozen suspension; Figure S5: Comparison between the IXS spectra measured at Q = 21 nm${}^{-1}$ on the ice sample and the frozen suspension; Figure S6: Comparison between the IXS spectra measured at Q = 7 nm${}^{-1}$ on the ice sample and the frozen suspension; Figure S7: Comparison between the IXS spectra measured at Q = 5 nm${}^{-1}$ on the ice sample and the frozen suspension.

## Sample Availability

Samples of the compounds are available from the authors.

## Abbreviations

The following abbreviations are used in this manuscript:

INS	Inelastic Neutron Scattering
IXS	Inelastic X-ray Scattering
FWHM	Full Width Half Maximum
DHO	Damped Harmonic Oscillator
PC	Phononic Crystals
