# Supplementary material for: The Effect of Embedded Nanoparticles on the Phonon Spectrum of Ice: An Inelastic X-ray Scattering Study
Source: Nanomaterials (Basel). 2023 Mar 1;13(5):918. doi: 10.3390/nano13050918 (PMC10005414; doi:10.3390/nano13050918)
Supplement: Supplementary file 1 [file nanomaterials-13-00918-s001.zip › nanomaterials-2175417-supplementary.pdf]

Supplementary Materials of Ice phonon spectra and Bayes inference: a gateway to a new understanding of terahertz sound propagation in water

Alessio De Francesco,[1,2,*] Luisa Scaccia,[3] Ferdinando Formisano,[1,2] Eleonora Guarini,[4] Ubaldo Bafile,[5] Dmytro Nykypanchuk,[6] Ahmet Alatas,[7] Mingda Lee,[8] Scott T. Lynch,[9] and Alessandro Cunsolo[10]

[1]*CNR-IOM & INSIDE@ILL c/o Operative Group in Grenoble (OGG), F-38042 Grenoble, France*

[2]*Institut Laue-Langevin (ILL), F-38042 Grenoble, France*

[3]*Dipartimento di Economia e Diritto, Università di Macerata, Via Crescimbeni 20, 62100 Macerata, Italy*

[4]*Dipartimento di Fisica e Astronomia, Università di Firenze, via G. Sansone 1, I-50019 Sesto Fiorentino, Italy*

[5]*Consiglio Nazionale delle Ricerche, Istituto di Fisica Applicata "Nello Carrara", via Madonna del Piano 10, I-50019 Sesto Fiorentino, Italy*

[6]*Brookhaven National Laboratory-National Synchrotron Light Source-NSLS II, P.O. Box 5000, Upton, 11973 NY, USA*

[7]*Argonne National Laboratory, Advanced Photon Source, P.O. Box 5000 Upton, 11973 NY, USA*

[8]*Department of Nuclear Science and Engineering, Massachusetts Institute of Technology, Cambridge, MA 02139*

[9]*Department of Physics, University of Wisconsin at Madison, 1150 University Avenue, Madison, WI, USA*

[10]*Department of Physics, University of Wisconsin at Madison, 1150 University Avenue, Madison, WI 53706, USA*

Abstract

As a contribution to the ongoing effort toward high-frequency sound manipulation in composite materials, we use Inelastic X-Ray Scattering to probe the phonon spectrum of ice, either in pure form or with a sparse amount of nanoparticles embedded in it. The study aims at elucidating the ability of nanocolloids to condition collective atomic vibrations of the surrounding environment. We observe that a nanoparticle concentration of about 1 % in volume is sufficient to visibly affect the phonon spectrum of the icy substrate, mainly canceling its optical modes and adding to it nanoparticle phonon excitations. We highlight this phenomenon thanks to a lineshape modeling based on Bayesian inference, which enables us to capture the finest detail of the scattering signal. The results of this study can empower new routes toward the shaping of sound propagation in materials through the control of their structural heterogeneity.

Figure S1 compares the spectra measured in the icy gold nanoparticle (Au-NP) suspension and in ice at $Q = 15$ nm^{-1}. Measured lineshapes are normalized as in Figure S1 of the paper's main body, yet compared without vertical offset. Furthermore, a vertical shadowed band roughly cover the spectral region dominated by the optical phonons of ice discussed throughout the main body of the paper. It is worth noticing that the case illustrated in Figure S1 is slightly more complex than those typically observed at different Q's. Although at this Q value and in the shadowed region of Figure S1 the Bayesian algorithm infers without ambiguity the presence of two inelastic modes in pure ice, its verdict is less unequivocal for the frozen suspension spectrum. Regardless, Figure S1 emphasizes the sizable effect that an even sparse amount of embedded Au NPs can have on the phonon response of ice. In fact:

1) If two inelastic modes are present in both spectra, these can be the same or different for the two samples. If the same, they appear visibly smothered and red-shifted in the NP spectrum. However, a difference is likely to exist, and this is the Au LFM$_G$ phonon in the suspension spectrum; its presence would make the shoulder on its right side the only discernible remnant of the optical doublet of ice.

2) The one-mode scenario would entail a more radical NP-induced transformation, i.e. the suppression of one of the two optical phonons of ice, or both of them if the only mode

* Correspondence email address: defrance@ill.fr

detected in the suspension spectrum by the fitting is the LFM_G phonon of gold, whose estimated frequency is 6.81 ± 0.08 meV.

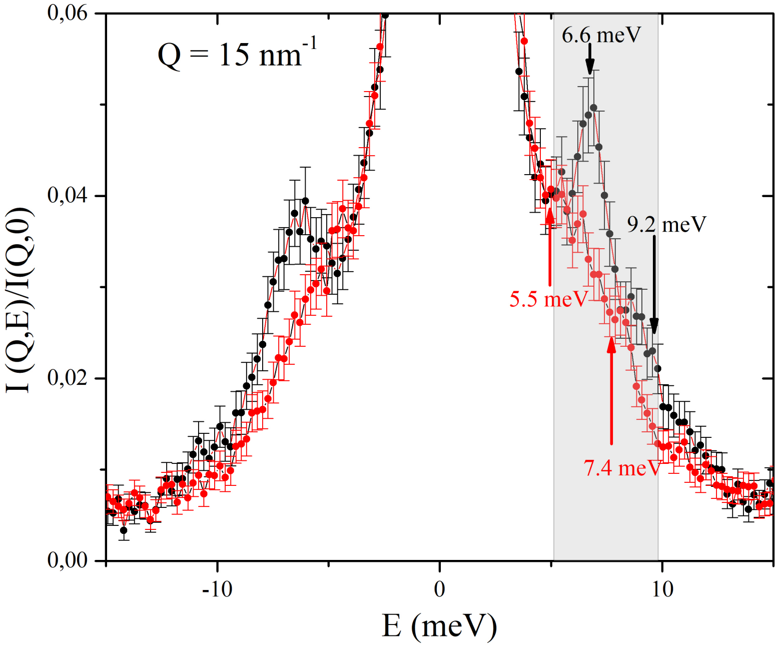

Figure S1. IXS spectra measured on polycrystalline ice (black dots) and AuNP frozen suspension (red dots) at $Q = 15$ nm^{-1} are compared in a restricted energy range after normalization to the respective maxima. The shadowed grey band delimits the zone where the optical modes of ice are detected. The two arrows pointing the red curve indicate the excitation frequencies when the suboptimal scenario is chosen from the Bayesian inference analysis.

Best-fitting model lineshapes and related DHO components corresponding with these two hypotheses are compared with the experimental spectrum in Figure S2. Both model options provide a consistent description of the rather broad inelastic shoulder on the side of the dominating central peak. Based on a mere visual inspection and our knowledge of the sample's phonon response, a clear-cut decision is far from obvious. This decision can undoubtedly benefit from examining the mode frequency posterior distribution drawn by the algorithm for the two competing hypotheses, which are compared in Figure S3. There it appears the single-mode option in orange and the double-mode one in wine.

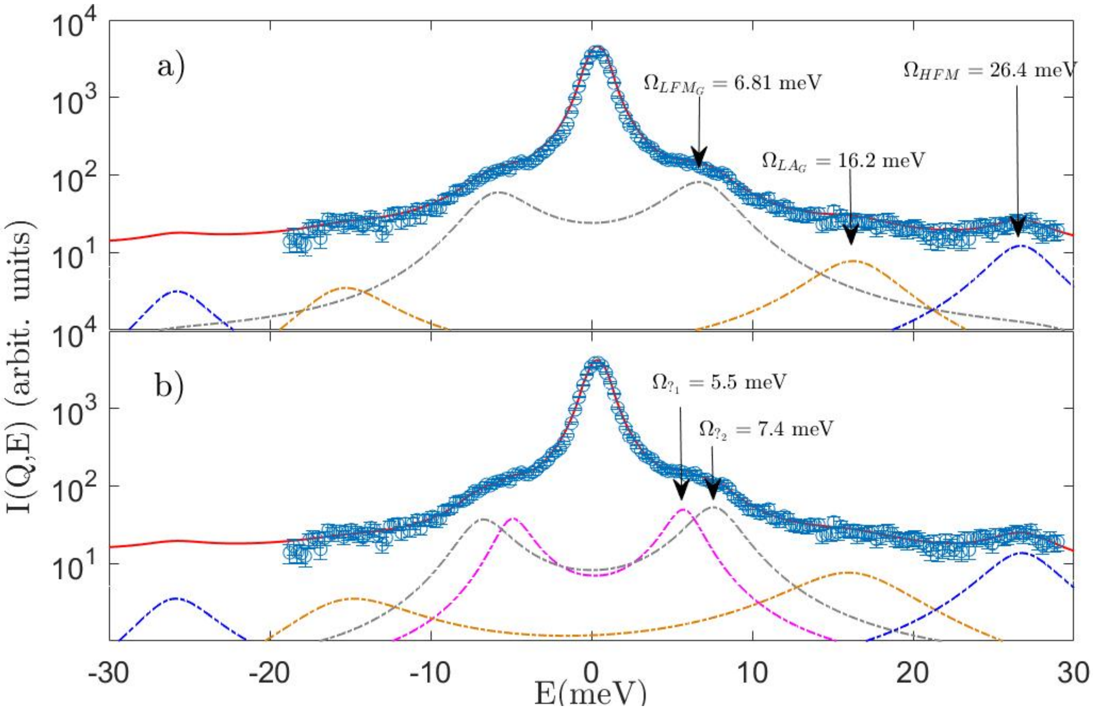

Figure S2. IXS spectrum of the AuNP frozen suspension (same Q of Figure S1) along with the best fit (red line) and its inelastic components (same color code as in the main manuscript) for the optimal solution (a) and the suboptimal solution (b) estimated by the MCMC-RJ algorithm. The question marks allude to the different possible interpretations mentioned in the text here above. The colors in the panel b) refer to hypothesis 1) here in the text.

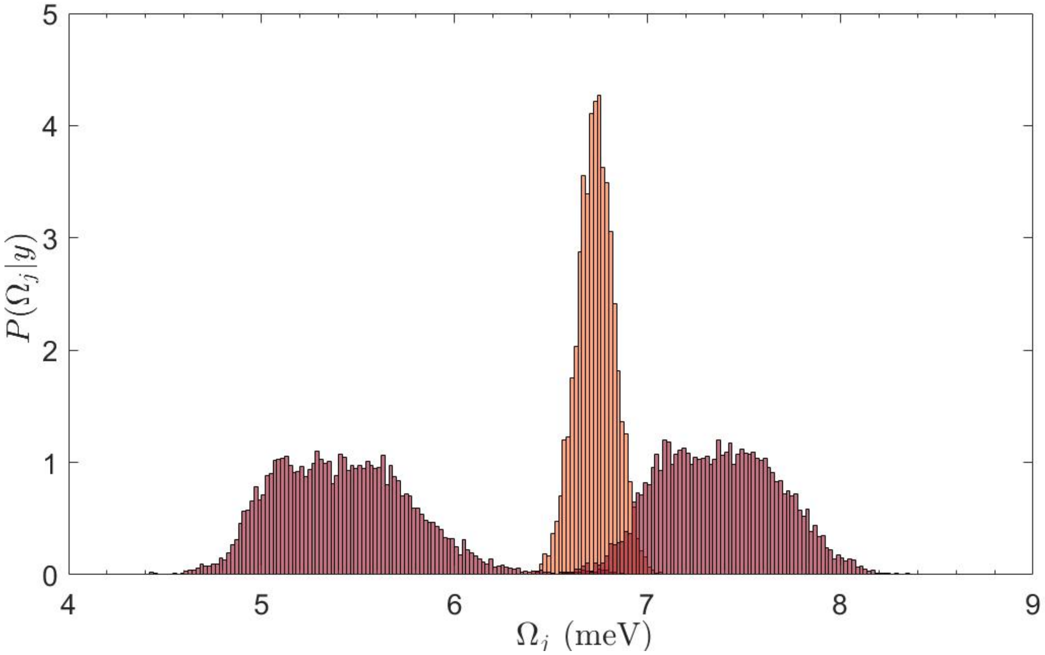

Figure S3. Posterior distribution functions for the excitation frequencies Ω_j in the low energy range, for the single mode found by the fitting algorithm as in panel a) of Figure S2 (grey line) and for the doublet as in panel b) of the same figure (magenta and gray lines). The colors of the distributions (see text) correspond to the hypothesis relating to the most probable model (single mode) and to the less probable one (double mode).

I. A MODEL FREE ASSESSMENT OF SCATTERING CONTRIBUTIONS FROM H_2O AND GOLD

A fair assessment of the NP influence on the ice dynamics requires a clear discernment of the spectral contributions from the two scattering media, H_2O, and gold. Given the difficulties associated with a rigorous evaluation of the Q dependent cross-section of the two samples, a viable alternative could be comparing the IXS signals collected from the samples in comparable conditions. Namely, the measurements should be executed with the same spectrometer setting, no more than a few days apart, with the same integration time, and on samples with the same shape and (illuminated) volume. Furthermore, sample containers should be identical and yield a negligible, or merely elastic, contribution to the total scattering signal. Since all these conditions were met in current measurements, a direct

comparison of the raw non-normalized signals measured from the two samples is significant. In Figure S4, spectral shapes measured in the two samples at 19 nm^{-1} are compared either in an expanded view (left panel) or in full scale (right panel). A rough inspection of the plots suggests that embedded Au-NPs significantly change the relative intensity and, likewise, the number of dominant inelastic features in the scattering profile. Indeed, one can readily appreciate the disappearance in the frozen suspension spectrum (red line+dot) of a few spectral features present in the ice one (black line+dot). Some of them are encircled by ellipses. Interestingly, the ice spectrum features a more prominent elastic peak. Still, the resolution-broadened quasielastic wings of such a peak (right plot) do not mask the presence of two well-resolved side peaks (left plot), which are absent in the frozen solution spectrum. In fact, in the left graph of Figure S4, it appears that (see larger ellipsis) the region covered by the lowest energy inelastic ice peaks appears nearly depleted in the suspension spectrum. The phenomenology outlined in Figure A is consistent with what was observed at other high Q values. For instance, one can look at Figure B, which compares the raw IXS spectra collected on the two samples at $Q = 21$ nm^{-1}. Notice that the spectra in Figure B are the same as in Figure S7 of the manuscript, yet displayed without normalization to the central peak intensity. Once again, the comparison suggests that embedded nanoparticles significantly attenuate the dominant phonon peak of ice (sitting at about 8 meV). Such attenuation is substantially more pronounced than that of the elastic peak, as demonstrated by Figure S7 of the manuscript, where, indeed, the two lineshapes are compared after normalization to the spectral intensity maximum. Consequently, the 8 meV ice phonon mode is attenuated more than the corresponding elastic peak. Hence, this attenuation is unlikely to stem uniquely from the more significant Au absorption, which would affect all spectral modes, elastic or inelastic, in equal measure.

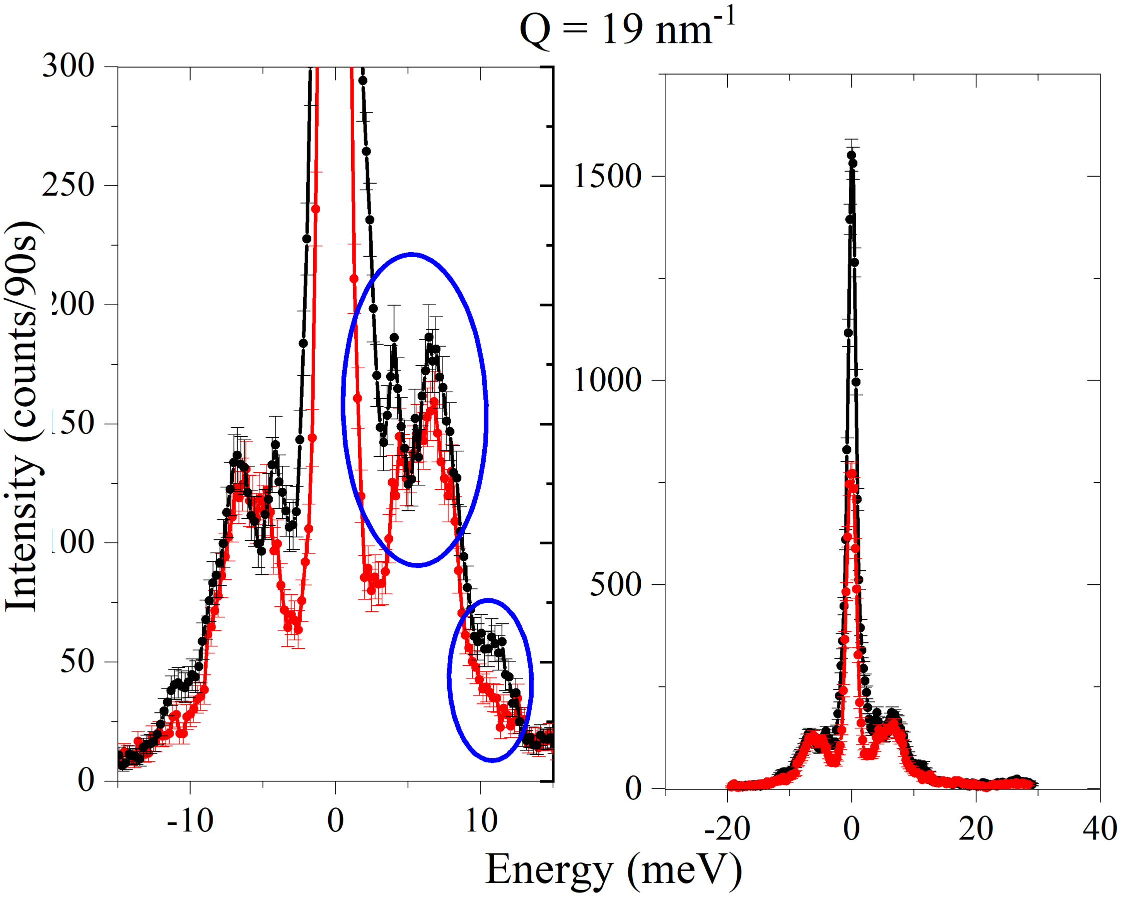

Figure S4. Left panel: comparison between the IXS spectrum measured at $Q = 19\ \mathrm{nm}^{-1}$ on the ice sample (black line-dot-line) and the frozen suspension (red line-dot-line). The spectral profiles are displayed in an expanded view, and the dashed ellipses encircle the region where differences between the phonon modes of the two samples are most evident. Right panel: the same comparison is reported in full scale.

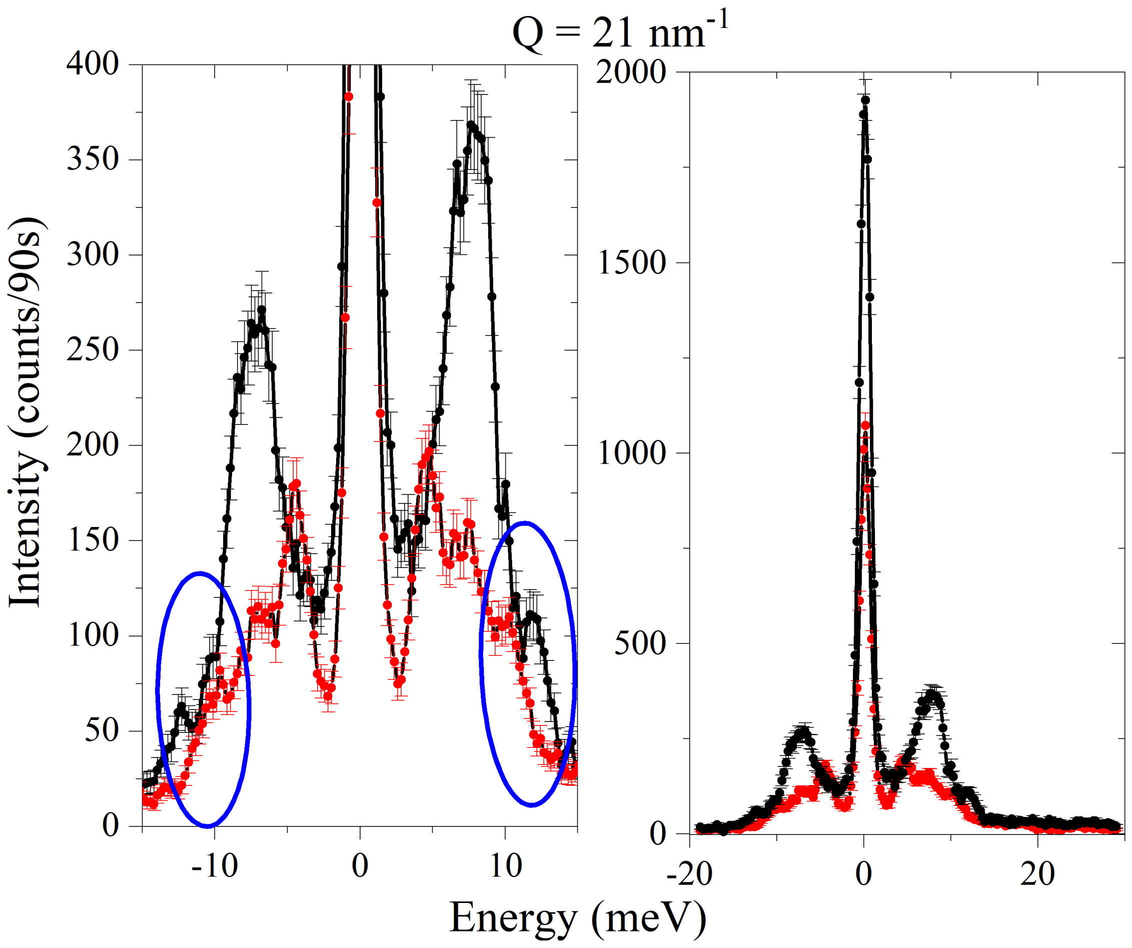

Figure S5. As in Figure S4, but for $Q = 21\ \mathrm{nm}^{-1}$.

In principle, one may still object that the comparison proposed thus far is not conclusive about the influence of NPs on the substrate dynamics due to two competing effects: the intensity suppression due to the strong Au absorption and its enhancement due to the large Au scattering. This scenario would suggest a preponderance of the Au scattering, yet it could hardly account for the phenomenology observed in Figures A and B. Furthermore, this scenario would collide with the low Q trend of the spectral shapes. To show this explicitly, in Figures S6 and S7, we compare the IXS spectral shapes measured on the two samples at 7 nm $^{-1}$ and 5 nm^{-1}, respectively. It appears that, in these lineshapes, ice phonons dominate the inelastic wings of both samples, albeit their relative amplitude becomes somehow depressed in the suspension spectrum at 5 nm^{-1}. Most importantly, at these low Qs, gold nanoparticles do not significantly contribute to the scattering profile of the suspension and do not significantly attenuate (absorb) the overall scattering profile. Here their leading effect seems to be a

selective attenuation of the ice phonons, especially at 5 nm^{-1}. Arguably, the nanoparticle elastic scattering at sufficiently low Qs should become preponderant, which indeed appeared to be the case of the $Q = 3$ nm^{-1} spectrum.

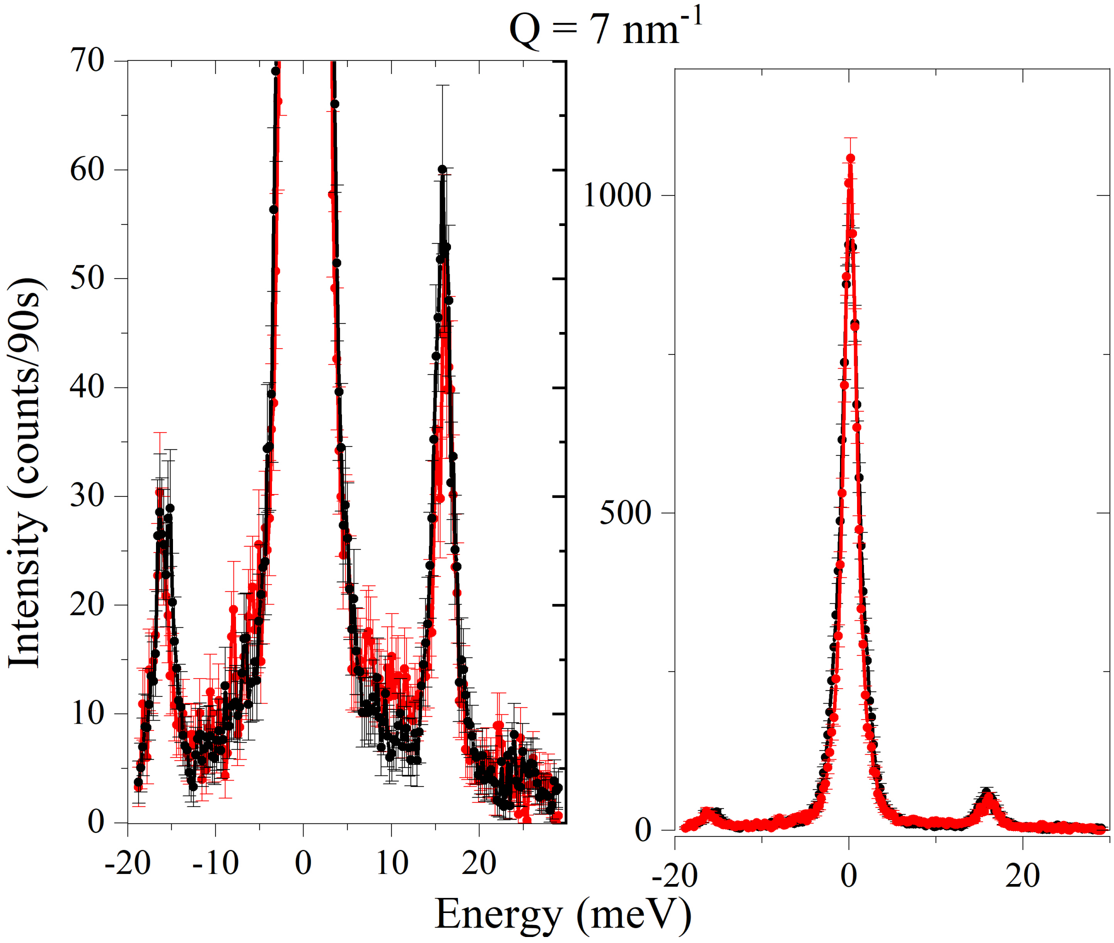

Figure S6. As in Figures S4 and S5, but for $Q = 7$ nm^{-1}.

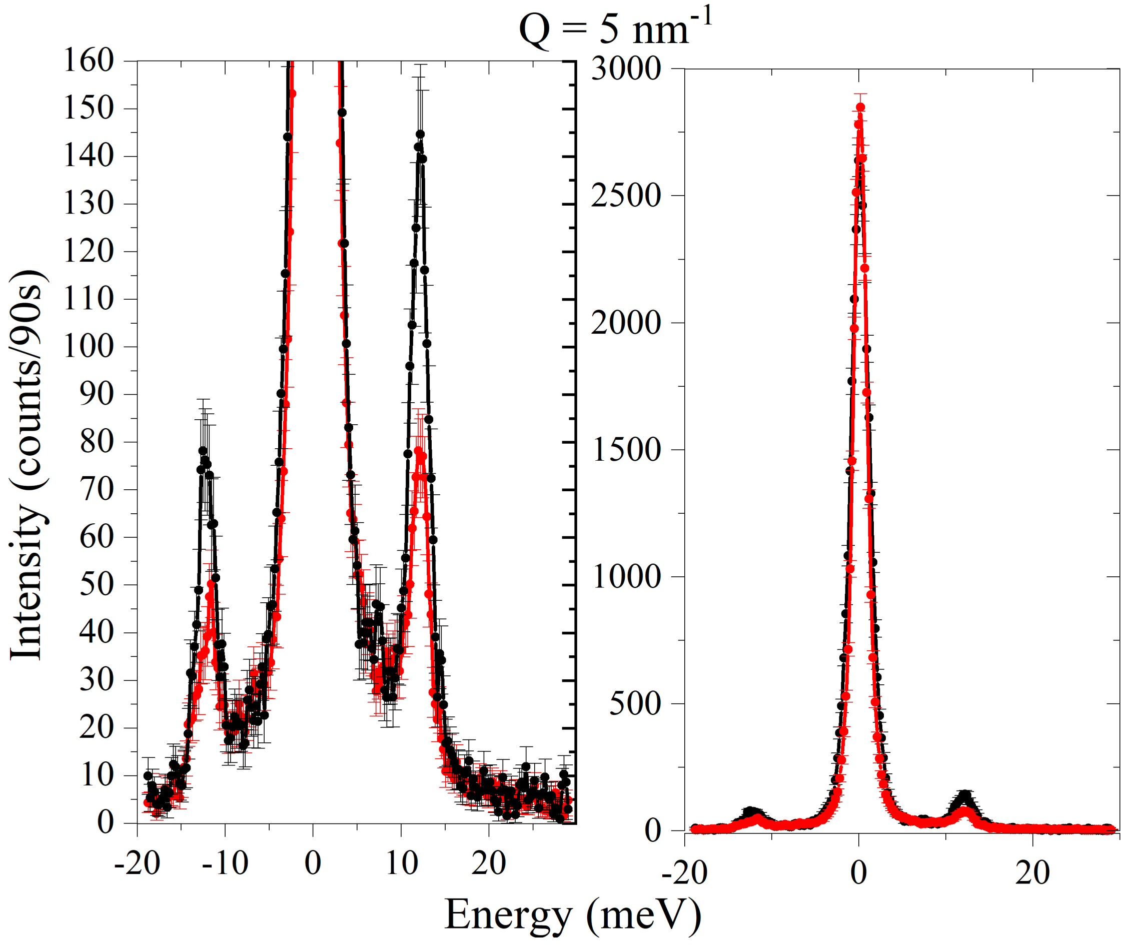

Figure S7. As in Figures S4, S5 and S6, but for $Q = 5\ \mathrm{nm}^{-1}$.
